# Altered expression of apoptosis‐related genes in rheumatoid arthritis peripheral blood mononuclear cell and related miRNA regulation

**DOI:** 10.1002/iid3.914

**Published:** 2023-07-12

**Authors:** Hamidreza Ebrahimian, Maryam Akhtari, Maassoumeh Akhlaghi, Elham Farhadi, Ahmadreza Jamshidi, Gholam Hossein Alishiri, Mahdi Mahmoudi, Mahmood Tavallaie

**Affiliations:** ^1^ Human Genetic Research Center Baqiyatallah University of Medical Sciences Tehran Iran; ^2^ Tobacco Prevention and Control Research Center (TPCRC), National Research Institute of Tuberculosis and Lung Diseases (NRITLD) Shahid Beheshti University of Medical Sciences Tehran Iran; ^3^ Rheumatology Research Center Tehran University of Medical Sciences Tehran Iran; ^4^ Inflammation Research Center Tehran University of Medical Sciences Tehran Iran; ^5^ Chemical Injuries Research Center, Systems Biology and Poisonings Institute Baqiyatallah University of Medical Sciences Tehran Iran; ^6^ Department of Rheumatology, Faculty of Medicine Baqiyatallah University of Medical Sciences Tehran Iran

**Keywords:** apoptosis, autoimmunity, BAX, FOXO1, miRNAs, P53, RB1, rheumatoid arthritis (RA)

## Abstract

**Aim:**

Impaired apoptosis and proliferation resulted in autoreactive lymphocyte development and inflammation in Rheumatoid arthritis (RA). *TP53*, *BAX*, *FOXO1*, and *RB1* are related genes in cell survival, proliferation, and inflammation which could be important in RA development and disease severity. Here we investigated their expression in peripheral blood mononuclear cells (PBMCs) from RA patients in comparison to healthy controls.

**Methods:**

Fifty healthy controls and 50 RA patients were selected. The quantitative real‐time polymerase chain reaction was used to assess the gene expression level in PBMCs.

**Results:**

The mRNA expression of *TP53* (FC = 0.65, *p* = .000), *BAX* (FC = 0.76, *p* = .008), *FOXO1* (FC = 0.59, *p* = .000) and *RB1* (FC = 0.50, *p* = .000) were significantly reduced in RA PBMCs. *TP53* expression was negatively correlated with miR‐16‐5p (*p* = .032) and *FOXO1* expression was negatively correlated with miR‐335‐5p (*p* = .005) and miR‐34a‐5p (*p* = .014). A positive correlation was seen between *TP53* expression and its downstream gene, *BAX* (*p* = .001). *FOXO1* expression was also negatively correlated with disease activity, DAS28 (*p* = .021).

**Conclusion:**

All selected genes have downregulated expression in RA PBMCs which could be correlated with RA pathogenesis by regulating apoptosis, cell survival, inflammatory mediator production, and proliferation. Due to the correlation of miR‐16‐5p, miR‐34a‐5p, and miR‐335‐5p with *TP53* and *FOXO1* expression in RA PBMCs, they could be used as future therapeutic targets.

## Graphical abstract

Impaired apoptosis and proliferation resulted in autoreactive lymphocyte development and ‎inflammation in rheumatoid arthritis (RA). Our results demonstrated that the expression of TP53, BAX, FOXO1, and RB1 genes are downregulated in RA PBMCs which could be correlated ‎with RA pathogenesis by regulating apoptosis, cell survival, inflammatory mediator ‎production, and proliferation. Due to the correlation of miR‐16‐5p, miR‐34a‐5p, and miR‐‎‎335‐5p with TP53 and FOXO1 expression in RA PBMCs, they could be one of the ‎regulatory mechanisms and RA treatment targets.

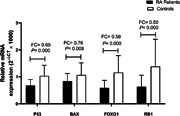

## 1 INTRODUCTION

Rheumatoid arthritis (RA) is a chronic inflammatory disease that primarily affects joints, but can also cause extra‐articular complications.^1^, ^2^ In general, both genetic and environmental factors (epigenetic) may contribute to the development of RA, including sex hormones, exposure to ultraviolet light, smoking, changes in the oral, lung, or intestinal microbiota, microbial infections, indeed the infections or tissue injury may be capable of reducing tolerance and inducing autoreactivity.^3^, ^4^, ^5^, ^6^ Synovial inflammation is closely related to changes in the function of RA fibroblast‐like synoviocytes (RAFLS). The FLSs' nonstop proliferation, cell survival, and production of pro‐inflammatory actors resulted in joint damage in RA.^7^

Programmed cell death also known as “controlled” cell death or apoptosis, is an important mechanism in maintaining organ homeostasis.^8^ It has been shown that cell proliferation and apoptosis were dysregulated in RA.^9^, ^10^, ^11^ Decreased FLS and lymphocyte apoptosis with aggressive proliferation and activation, interrupting joint and immune balance and function in RA patients.^12^, ^13^ Altered gene expression and genetic polymorphisms in apoptotic or cell cycle‐related genes have been reported in RA.

Approximately 70% of the human genome encodes the non‐protein‐coding genes including miRNA which play an essential role in genome expression and autoimmune pathogenesis as an epigenetic factor.^14^ miRNAs are evolutionarily conserved with 18–25 nucleotides, which have an essential function in gene expression.^15^ Many studies reported the critical role of miRNAs in autoimmune diseases such as RA.^15^, ^16^, ^17^, ^18^

Based on our last experiment miR‐335‐5p, miR‐150‐5p, miR‐34a‐5p, and miR‐16‐5p were upregulated in peripheral blood mononuclear cells (PBMCs) of RA patients. In the miRTaRBase database (https://mirtaRBase.cuhk.edu.cn/~miRTaRBase/miRTaRBase_2022/php/index.php), these miRNAs target the expression of important genes contributing to the cell‐cycle and apoptosis regulation. The miR‐335‐5p targeted *RB1*, and *FOXO1*, the miR‐150‐5p targeted *TP53*, the miR‐34a‐5p directly targeted *BAX*, and *TP53*, and indirectly targeted *FOXO1*, and the miR‐16‐5p targeted *TP53*.^19^

P53 tumor suppressor protein which is referred to as the “Guardian of the Genome” is encoded by the *TP53* gene and is one of the most important proteins that contributes to cell‐cycle regulation.^20^ P53 protein protects the DNA integrity in cells, however, it is also involved in other functions such as aging, cell differentiation, and development.^21^ P53 abnormality resulted in cell survival and nonstop proliferation alongside DNA damage.^22^ P53 abnormal function or dysregulation was reported in RA synovial cells or lymphocytes which led to DNA damage and resistance to apoptosis.^23^, ^24^ Forkhead box protein O1 (FOXO1), is a protein encoded by the *FOXO1* gene. FOXO1 is also involved in important pathways including apoptosis, cell cycle, oxidative stress response, autophagy, immune system regulation, DNA repair, cell differentiation, and metabolism.^25^, ^26^, ^27^, ^28^ *FOXO1* dysregulation is reported in different diseases including RA.^29^, ^30^ The RB1 is encoded by the *RB1* gene which was discovered in the malignant tumor of the retina known as the retinoblastoma.^31^ The tumor suppression activity of RB1 was well‐defined in the inhibition of cell cycle progression.^32^, ^33^ Alongside cancers, *RB1* dysregulation was reported in RA pathogeneses.^34^, ^35^ Apoptosis regulator BAX, BCL2 associated X, was encoded by the *BAX* gene. The main function of this protein is initiating the apoptosis pathway by releasing proapoptotic factors, such as cytochrome c from mitochondria.^36^ The importance of autoreactive lymphocyte survival and apoptosis resistance was one of the main factors in autoimmunity pathogenesis. The low expression level of *BAX* was reported in RA lymphocytes which reduces the apoptosis in these cells.^11^ The correlation of the expression of mentioned miRNAs with the expression of targeted genes has previously been confirmed in targeting these genes by strong evidence.

Herein, we investigate the expression of *RB1*, *BAX*, *FOXO1*, and *TP53* which are targets of mentioned miRNA and are essential genes in cell cycle progression and apoptosis in PBMCs from Iranian RA patients and also analyze the correlation between the expression of these genes with their corresponding miRNAs.

## 2 METHODS

### 2.1 Study subjects

Fifty patients with RA diagnosis were selected according to the American College of Hematology criteria^37^ and recruited to the Rheumatology Research Center, Shariati Hospital, Tehran, Iran. Fifty matched controls in age and gender were also selected with not any autoimmunity diagnosis even in their relatives. The human research ethics committee of Tehran University of Medical Science reviewed and approved the current study (Approval No: ‎ IR.TUMS.VCR.REC.1398.885).

### 2.2 Measuring transcription levels of *RB1*, *TP53*, *BAX*, and *FOXO1*

For mRNA expression analysis first‐strand cDNA was synthesized by RT‐ROSET (REF: EB983019, ROJE Technologies). The Real Q Plus Master Mix Green High ROX (AMPLIQON) was used for quantitative real‐time polymerase chain reaction (PCR) (Step One Plus, Applied Biosystems). The test reaction included 10 μL 2× SYBR Green PCR master mix, 8 μL cDNA (1/8 dilution), and 1 μL each primer for reaction. The primer details are listed in Table 1. Real‐time PCR was conducted as follows: 95°C for 15 min, 95°C for 15 s, 56°C for 30 s, and 72°C for 1 min. For normalizing the relative quantification levels of the target mRNAs, *GAPDH* mRNA transcript level was used. Livak and Schmittgen's method was used in relative mRNA expression calculation.^38^

**Table 1:** Primer sequences and details.

Genes	Primer set sequence	Tm (°C)	Amplicon size (kb)	References
*FOXO1*	5ʹ‐GCAGATCTACGAGTGGATGGTC‐3ʹ (forward primer)	60.29	325	[30]
	5ʹ‐AAACTGTGATCCAGGGCTGTC‐3ʹ (reverse primer)	60.27		
*RB1*	5ʹ‐ACCAGATCATGTCAGAGAGA‐3ʹ (forward primer)	54.97	340	[39]
	5ʹ‐ACTGCTGGGTTGTGTCAAAT‐3ʹ (reverse primer)	57.93		
*BAX*	5ʹ‐GGACGAACTGGACAGTAACATGG‐3ʹ (forward primer)	61.17	150	[40]
	5ʹ‐GCAAAGTAGAAAAGGGCGACAAC‐3ʹ (reverse primer)	60.85		
*TP53*	5ʹ‐TCAACAAGATGTTTTGCCAACTG‐3ʹ (forward primer)	58.57	118	[41]
	5ʹ‐ATGTGCTGTGACTGCTTGTAGATG‐3ʹ (reverse primer)	61.39		
*GAPDH*	5ʹ‐AAGGTCGGAGTCAACGGATTT‐3ʹ (forward primer)	59.65	66	[42]
	5ʹ‐ACCAGAGTTAAAAGCAGCCCTG‐3ʹ (reverse primer)	60.82		

### 2.3 Statistical analysis

SPSS v.28 was used to calculate data. The Kolmogorov–Smirnov test was applied for For normality evaluation. Spearman's rho test or Pearson's correlation coefficient was carried out for correlation analysis. For gene expression analysis Unpaired *T* test or Mann–Whitney were performed. The GraphPad Prism v.8 software was used for plotting.

## 3 RESULTS

### 3.1 Study groups

Clinical details of healthy control and RA patients are listed in Table 2. The disease‐modifying antirheumatic drugs (DMARDs), including methotrexate (MTX), hydroxychloroquine, prednisone (steroid), sulfasalazine, and alendronate were in RA patients' treatment program. Six patients are in early diagnosis and do not take any treatment.

**Table 2:** Clinical characterization of RA patients and control.

Item	RA patients (*N* = 50)	Control (*N* = 50)
Age	48.54 ± 11.54	47.24 ± 9.85
Gender (*N*)	Female (43)/male (7)	Female (43)/male (7)
Treatment duration	38.30	
Drug	Prednisone (36), methotrexate (29), hydroxychloroquine (16), sulfasalazine (13), alendronate (13)	No drugs
DAS28	5.10 ± 1.2	‐
Family history	RA (9), DM (10), SLE (1), MS (2), thyroid diseases (3)	No diseases
Concurrent syndrome	thyroid diseases (6), DM (4)	No diseases
RF (%)	33 (66)	‐
ACCP (%)	43 (86)	‐

Abbreviations: ACCP, anti‐cyclic citrullinated peptide; DAS28, Disease Activity Score of 28 joints; DM, diabetes mellitus; MS, multiple sclerosis; RA, rheumatoid arthritis; RF, rheumatoid factor; SLE, systemic lupus erythematosus.

### 3.2 Evaluation of *TP53, BAX, FOXO1*, and *RB1* expression

**Figure 1:**
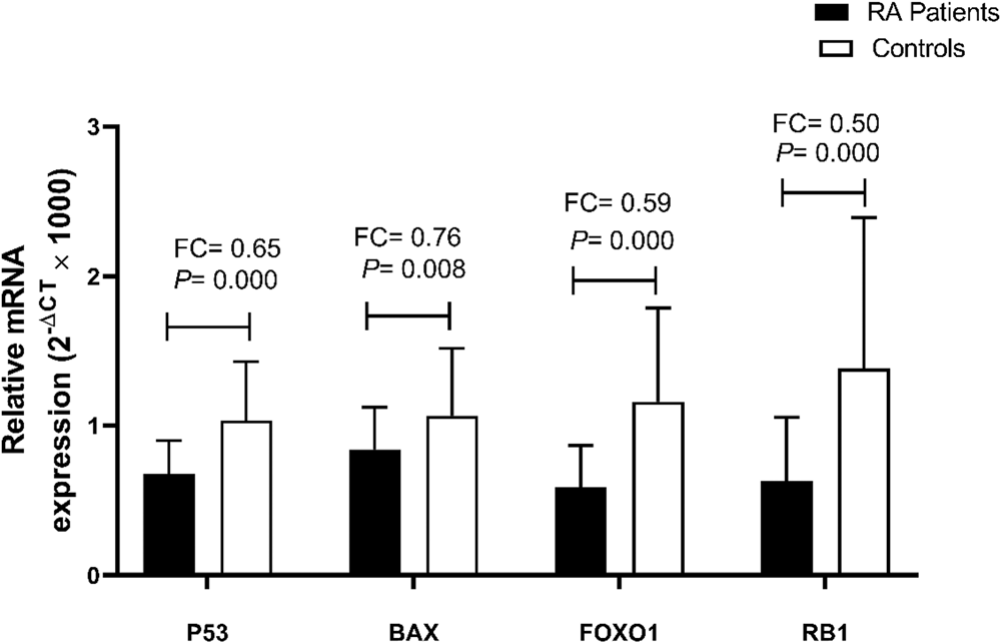
*TP53*, *BAX*, *FOXO1*, and *RB1* expression in RA patients and control. The expression was assessed by real‐time PCR. The groups were compared by the Mann–Whitney test. All expressions show a significant reduction in RA patients. RA, rheumatoid arthritis.

The mRNA expression of *TP53* (FC = 0.65, *p* = .000), *BAX* (FC = 0.76, *p* = .008), *FOXO1* (FC = 0.59, *p* = .000), and *RB1*(FC = 0.50, *p* = .000) were significantly reduced in PBMCs of RA patients in comparison to controls (Figure 1). No correlation was found between prednisone, methotrexate, hydroxychloroquine, and sulfasalazine with all gene expression in RA patients. In RA patients with alendronate treatment *TP53* expression was significantly upregulated (*p* = 039) and *RB1* expression downregulated (*p* = .032). however, the mRNA expression of TP53 and RB1 was also significantly decreased ‌(*p* = .000 and *p* = .001, respectively) in RA patients who did not receive alendronate treatment compared to controls.

### 3.3 Correlation analysis

Based on our recent experiment,^42^ the miRNA expression levels of miR‐335‐5p, miR‐16‐5p, miR‐150‐5p, and miR‐34a‐5p were considerably higher in patient‐derived PBMCs than in control samples. In the present work, we explored the relationship between the expression of the analyzed genes and these four miRNAs in RA patients' PBMCs. *TP53* expression was negatively correlated with miR‐16‐5p expression (*p* = .032) and *FOXO1* expression was also negatively correlated with miR‐335‐5p expression (*p* = .005). The expression of miR‐34a‐5p was negatively correlated with *FOXO1* (*p* = .014). A positive correlation was seen between the expression of *TP53* and its downstream gene, *BAX* (*p* = .001). *FOXO1* expression was also negatively correlated with disease activity DAS28 (*p* = .021). All of the miRNAs' data were listed in Table 3.

**Table 3:** Correlation of the expression of miRNAs and *TP53*, *BAX*, *FOXO1*, and *RB1* in RA patients.

Genes	miR‐335‐5p	miR‐16‐5p	miR‐34a‐5p	miR‐150‐5p
*TP53* (*p* Value)	–0.196 (0.18)	−0.316 (0.032)*	−0.33 (0.82)	0.132 (0.38)
*BAX* (*p* Value)	−0.454 (0.001)*	−0.251 (0.088)	0.003 (0.98)	0.015 (0.92)
*FOXO1* (*p* Value)	−0.402 (0.005)*	−0.268 (0.049)*	−0.364 (0.014)*	0.241 (0.11)
*RB1* (*p* Value)	−0.260 (0.078)	−0.015 (0.92)	−0.075 (0.62)	−0.102 (0.51)

*Note*: Significant correlations were tagged by *.

## 4 DISCUSSION

RA is a systemic inflammatory autoimmune disease marked by the deterioration of joints.^43^ Apoptosis resistance and hyperproliferation in synovium tissue, RAFLS, and immune cells result in the creation of pannus and the inflammation and degeneration of joints.^44^ The development and selection of lymphocytes are closely regulated mechanisms that eliminate autoreactive cells by apoptosis. Due to autoreactive lymphocyte activity, any interruption of the cell cycle and survival leads to a variety of autoimmunity.^45^, ^46^ This notion is supported by lpr or gld mutations (mutations in the Fas and Fas ligand genes) in mice that generated autoantibodies^47^, ^48^ FasL was the inducer of apoptosis which induce clustering of Fas to trigger apoptosis signaling.^49^

In our previous report,^42^ we found a significant upregulation of some miRNAs including miR‐335‐5p, miR‐16‐5p, miR‐150‐5p, and miR‐34a‐5p in PBMCs of Iranian RA patients. Herein, the expression of their important target genes including *TP53*, *BAX*, *FOXO1*, and *RB1* which contribute to the cell‐cycle regulation was investigated in the same RA patients' samples. As we discussed later, the P53 and its downstream proteins including BAX, FOXO1, and RB1 were necessary for cellular apoptosis and proliferation which are essential in autoimmune disorders including RA. P53 was reported to be downregulated in mRNA and protein levels in different cell types including PBMCs^50^ and FLS^51^‌ in RA. Dysregulated expression or mutation of *TP53* which finally leads to abnormal cell function could enhance the aggressive nature of RAFLS or autoreactivity in lymphocytes and leads to the inflammatory phenotype in RA joints.^52^ *TP53* knockdown in RAFLS resulted in the production of inflammatory cytokines and T‐cell differentiation which is important in RA pathogenesis.^53^ *TP53* knockout mice were also reported to have higher arthritis scores compared with *TP53* ^+^ mice.^54^ *TP53* downregulation can cause dendritic cells (DCs) maturation and activation, and reduce the expression of *FOXP3* in T cells, which is a master regulator of regulatory T cells (Tregs) and Th17 polarization. All these changes were important in RA pathogenesis.^23^

Our study showed a significant reduction in *TP53* transcription in RA patients' samples that might be correlated with RA pathogenesis. One of the important regulators of this protein is miR‐16‐5p which significantly increased^42^ and negatively correlated with *TP53* expression in RA PBMCs. As much as we know, these findings are the first report in RA PBMCs. P53 also has the ability to induce the expression of proapoptotic genes such as *BAX*.^55^, ^56^ Herein, we reported the positive correlation between the expression of *TP53* and *BAX* in RA patients as one of the *BAX* regulatory mechanisms. In this study, a significantly lower expression of *BAX* was reported in RA PBMCs. It was previously reported that T lymphocytes in RA patients were resistant to apoptosis which could be correlated with reduced expression of some related genes including *BAX* in these cells.^11^ The induction of *TP53* and *BAX* expression was reported in another study which could help control normal apoptosis as a new strategy for the treatment of RA.^57^

FOXO1 is an important mediator in apoptosis, autophagy, antioxidative stress, cell cycle arrest, metabolism, and immune system regulation, which was important in the pathogenesis of different diseases.^58^ Previous studies reported a lower expression level of *FOXO1* in PBMCs of RA patients which could be correlated with disease pathogenesis and severity.^30^ The other study reported that lower expression of *FOXO1* in patients' peripheral blood and synovial tissue is correlated with disease pathogenesis, clinical parameters, and inflammatory mediators such as IL‐6 expression.^29^ Another study revealed that *FOXO1*‐silenced RAFLSs have increased survival with reduced apoptosis due to a reduction in caspase 3/7 activity.^59^ *FOXO1*‐silenced RAFLSs also have reduced inflammatory cytokines including IL‐6, IL‐8, TNF‐α, and IL‐1β which were essential in RA pathogenesis.^59^ Herein, we observed the lower expression of *FOXO1* in PBMCs of RA patients which also negatively correlated with disease activity, DAS28 in patients. Our previous study reported the significant upregulation of miR‐34a‐5p and miR‐335‐5p.^42^ It was reported that miR‐34a‐5p and miR‐335‐5p were negatively correlated with *FOXO1* expression in the RA background.

In general, the cell cycle is progressed with cyclin‐cyclin‐dependent kinase (cyclin‐CDK). RB1 is inactivated with CDK kinase activity. Cyclin‐dependent kinase inhibitors (CDKIs) activated the RB1 by inhibiting CDK activity. It was reported that CDKI gene delivery into arthritic joints could arrest RAFLS proliferation and reduce the expression of pro‐inflammatory cytokine.^60^ Active RB1 transfer to RAFLS suppresses the matrix metalloproteinase‐3 (MMP‐3) and monocyte chemoattractant protein‐1 (MCP‐1) production which was crucial in inflammation and joint destruction in RA.^60^ *TP53* induction in RAFLS resulted in P21 overexpression, a potent inhibitor of CDK, and could activate RB1 and cell cycle progression in these cells.^51^ Herein, the low expression level of *RB1* was reported in PBMCs of RA patients which might be important in the pathogenesis of this disorder.

Based on our analysis the reduction in the expression of studied genes in patients was not associated with their received treatment and we did not find any significant association between patients' treatment and the expression of selected genes, except for alendronate. Alendronate was able to alter *TP53* and *RB1* gene expression to some extent in PBMCs of RA patients that did not influence the statistical analysis.

In conclusion, the expression of apoptosis and cell survival‐related genes, such as *TP53*, *BAX*, *FOXO1*, and *RB1*, was reduced in RA patients' PBMCs. This reduction in gene expressions may result in cell survival, resistance to apoptosis, and expression of inflammatory mediators, which were crucial for RA joint destruction or autoreactive lymphocyte selection. Here, the association between the expression of these genes and the miRNAs previously identified to target them was explored in RA PBMCs. Based on our results, miR‐16‐5p may negatively influence the expression of *TP53*, while miR‐335‐5p and miR‐34a‐5p negatively regulate the expression of *FOXO1* in RA PBMCs. Due to the fact that there is no specific and effective treatment for RA patients, these regulatory miRNAs and their target genes or molecular pathways might be used as future therapeutic targets which require additional research.

## AUTHOR CONTRIBUTIONS

*Acquisition of data, drafting the article, analysis and interpretation of data, and final approval of the article*: Hamidreza Ebrahimian, Maryam Akhtari, Maassoumeh Akhlaghi, and Gholam Hossein Alishiri. *The conception and design of the study, revising the article critically, interpretation of data, and final approval of the article*: Elham Farhadi, Ahmadreza Jamshidi, Mahdi Mahmoudi, and Mahmood Tavallaie.

## CONFLICT OF INTEREST STATEMENT

The authors declare no conflict of interest.

## ETHICS STATEMENT

This study was performed based on the Declaration of Helsinki guidelines and‎ was approved by ‎the Ethics Committee of Tehran University of Medical Sciences (Approval No: ‎ IR.TUMS.VCR.REC.1398.885). The written informed consent was signed by all participants before enrolling in the study.

## Data Availability

The data generated and/or analyzed during the current study are available from the ‎corresponding author upon reasonable request.‎
